# Ultrasonographic Evaluation of the Patellar Tendon in Cyclists, Volleyball Players, and Non-Practitioners of Sports—The Influence of Gender, Age, Height, Dominant Limb, and Level of Physical Activity

**DOI:** 10.3390/jfmk9030116

**Published:** 2024-07-01

**Authors:** Ângela Pissarra, Luís Ribeiro, Sónia Rodrigues

**Affiliations:** 1 Medical Imaging and Radiotherapy, School of Health, University of Algarve, 8005-139 Faro, Portugal; angepatricia@sapo.pt (Â.P.); srodrigues@ualg.pt (S.R.); 2 Research Unit for Sport and Physical Activity, Faculty of Sport Sciences and Physical Education, University of Coimbra, 3004-531 Coimbra, Portugal; 3 SPRINT, Sport Physical Activity and Health Research & Innovation Center, 2001-904 Santarém, Portugal; 4 Centre for Health Studies and Development, School of Health, University of Algarve, 8005-139 Faro, Portugal

**Keywords:** patellar tendon, measurements, ultrasound, sport, cyclists, volleyball players

## Abstract

This study was based on the ultrasound evaluation of the patellar tendon dimensions in the anteroposterior and transversal approaches in sports practitioners (cyclists and volleyball players) and non-sports practitioners. Relationships between the patellar tendon length, width, and thickness with gender, age, height, the dominant limb, and level of physical activity were evaluated. The samples included cyclists, volleyball players, and non-sports practitioners between 15 and 25 years old. Individuals were positioned supine with 30 degrees of knee flexion for bilateral measurements. Sports practitioners presented with an increased patellar tendon length and thickness. There were significant differences bilaterally between sports and non-sports practitioners (*p* < 0.003). The three dimensions of the patellar tendon of both limbs presented correlations with the male gender (0.336 < r < 0.601), and values of moderate-to-strong intensity in the length of the patellar tendon was directly proportional to height (0.520 < r < 0.601). There was a significant difference between the patellar tendon width and age (*p* < 0.025). Regarding the level of physical activity, significant differences were found between low and high levels in the three dimensions bilaterally (*p* < 0.004). The study results indicate that gender, age, height, and level of physical activity impact patellar tendon dimensions. However, there are no associations with the dominant limb.

## 1. Introduction

The patellar tendon (PT) is a connective tissue structure with a width between 2.7 and 3.8 cm proximally and 2.4 and 3.5 cm distally. Its thickness is 3 mm proximally and 5 mm distally, but it depends on the area, centrally 4.9 ± 0.4 mm, medially 4.4 ± 0.4 mm, and laterally 4.3 ± 0.6 mm, being thinner in women. Its length is 3.8–5 cm, with no gender differences [1,2].

This tendon changes its physical and chemical properties because of numerous factors, such as age, gender, height, temperature, hormonal influences, and sports activity [1,3]. The tendons and ligaments are more flexible and viscous up to the age of twenty, after which the musculoskeletal system matures [2,3,4,5]. The PT has an increase in length and thickness in males since the average height is higher due to hormonal differences between genders [6,7]. In addition, individual anthropometric variables, such as height, can predict an increase in the PT length and diameter. This relationship is attributed to growth and development processes [8].

Tendons are sensitive to training as they adapt to loads during exercise by increasing the size of collagen fibers [1,2]. However, PT injuries are common in sports practitioners (SPs), namely cycling and volleyball, since this tendon has greater tensile stress in these sports [1]. In volleyball, knee flexion movements are performed during the gait, swing, and support phases, with eccentric muscle action and subsequent concentric contraction in the impulse phase, increasing the tendon’s resistance [2,9]. As for cycling, an increasing knee flexion increases compressive stress. During the pedaling cycle, the knee undergoes flexion accompanied by medial rotation of the tibia of approximately 13 degrees [10]. On the other hand, in non-sports practitioners (NSPs), joint immobilization leads to muscle atrophy, causing a deterioration in the tendons and ligaments, which causes an overall loss of the strength and stiffness of these structures [1]. Therefore, this study will compare the PT measurements among those who practice and do not practice sports.

To evaluate PT measurements, we chose ultrasonography because it is an imaging technique that makes it possible to characterize and assess the PT at a low cost, it is non-invasive, it has no ionizing radiation, it is portable, and it is available. On the other hand, it has high spatial resolution due to the high-frequency transducer and allows for a contralateral comparison.

Ultrasonography allows for the diagnosis of tendinosis areas with the degeneration and disorganization of collagen fibrils, the hypoechoic thickening of the deep fibers of the PT. Also, doppler ultrasound can detect regions of neovascularization present in the PT of symptomatic patients [3]. The most common sonographic findings in athletes with patellar tendinopathy are hypoechoic thickening, intra-tendinous calcific deposits, enthesopathy at the inferior pole of the patella, abnormalities in the tendon–bone interface, and hypervascularization usually originating from the underlying Hoffa fat pad and invading the degenerated tendon. All of the aforementioned sonographic signs are crucial to planning a suitable rehabilitation approach, such as stretching, eccentric exercises, tendon load management, and ultrasound-guided procedures [11].

Therefore, the main objectives of this study were to verify if PT dimensions increase with the sports practice of cycling and volleyball and to understand if PT dimensions are influenced by gender, height, age, the dominant limb, and the level of physical activity (LPA).

This way, a correlational quantitative study was carried out to evaluate PT dimensions through ultrasound. Its anteroposterior and transversal approaches were assessed in SPs and NSPs, and the measurements of the PT—length (LPT), right (R) and left (L), width (WPT), and thickness (TPT)—were defined as dependent variables. The independent variables were gender, age, height, the dominant limb, and LPA.

## 2. Materials and Methods

### 2.1. Sampling

This study complied with people who met the following inclusion criteria: females and males between 15 and 25 years old who practiced cycling or volleyball or did not practice sports.

Our study included 115 participants: 51 females and 64 males, grouped into three groups: 40 NSPs, 37 cyclists, and 38 volleyball players. From this sample, 69 participants had a “high” LPA, with 6 “moderate”, and in the NSP group, 40 had a “low” level. All participants with acute lower limb symptoms and a history of knee surgery or pathology were excluded during the selection process.

### 2.2. Study Design

The instruments used were an ultrasound LOGIC 5 Expert from General Electric, a linear transducer, a goniometer, and the “International Physical Activity Questionnaire” (IPAQ). The World Health Organization developed this questionnaire to assess the physical activity levels of individuals in their daily lives using 27 questions, and it is available at https://sites.google.com/view/ipaq/download (accessed on 21 November 2021).

It is divided into five sections: physical activity at work, physical activity as transportation, physical activity at home (work, domestic tasks, and taking care of the family), physical activity recreation, sport, exercise, and leisure, and finally, time spent sitting. In addition to sociodemographic data, this questionnaire asks for details about the specific types of activities carried out in each domain, providing a score for walking, moderate-intensity activity, and vigorous-intensity activity. Finally, through calculations, subjects’ LPA is classified as “Low”, “Moderate”, or “High”.

### 2.3. Data Collection

**Figure 1:**
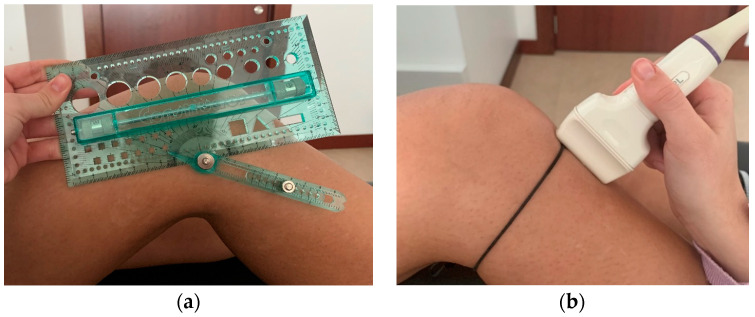
Position of the subject and the ultrasound transducer (**a**) knee flexion at 30 degrees using a goniometer; (**b**) hypoechoic wire around the PT region to the section length measurement.

To perform the ultrasound, the subjects were placed supine with their knee flexed at 30 degrees and the muscles relaxed. The correct knee angulation was ensured using a goniometer (Figure 1a) [12,13].

The ultrasound was displayed in B mode with a 10 MHz frequency, and the linear transducer was selected. Lateromedial scanning started in patients with tendons longer than 5 cm, and the transducer footprint sectioned the measurement of LPTR and LPTL by placing a hypoechoic wire around the knee in the tendon region (Figure 1b).

**Figure 2:**
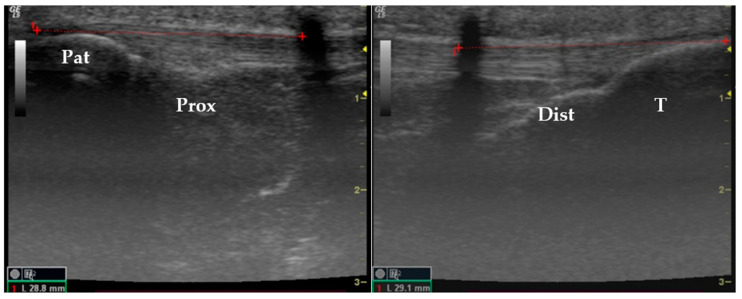
Longitudinal section of an ultrasound image showing the length measurement of the patellar tendon. Pat: patella; T: tibia; Prox: proximal; Dist: distal.

The PT length in these cases was quantified from its insertion in the patella to the wire and then from the wire to its insertion in the tibia (Figure 2).

The hypoechoic shadow observed on the ultrasound images is related to the acoustic shadow originating from the wire used to perform the investigation. Posterior acoustic shadowing is an artifact that occurs when ultrasound beams are reflected or absorbed during wave propagation, such as hypo or anechoic bands or regions, in this case, the wire.

The researcher performed scans and measurements of the PT. These measurements were repeated three times to reduce intra-operator variability.

**Figure 3:**
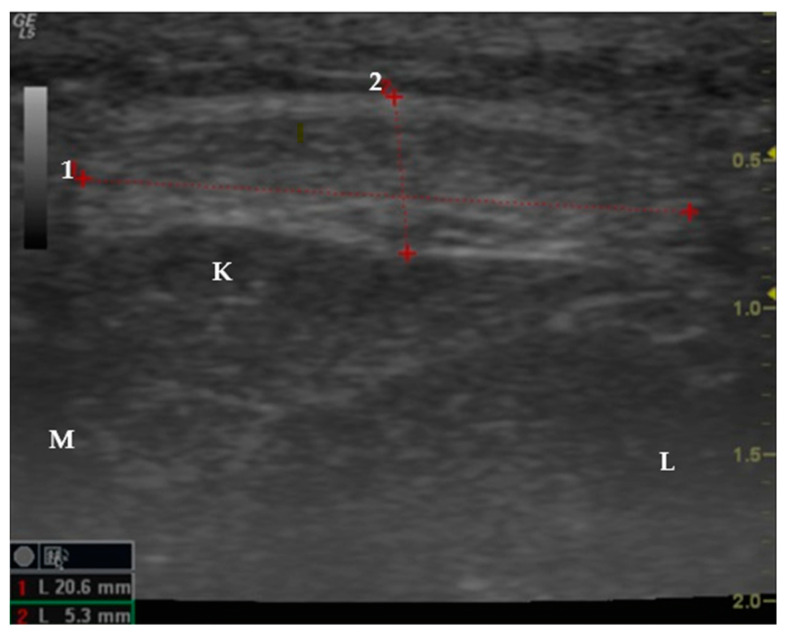
Transversal section of an ultrasound image showing the width (1) and thickness (2) measurements of the patellar tendon. K: Kager’s fat pad; L: lateral; M: medial.

Subsequently, the transducer was rotated 90 degrees for a cross-sectional view to proceed with craniocaudal scanning. On this axis, we simultaneously obtained two measurements: width (1) and thickness (2) (Figure 3). 

The process was repeated three times, and the results were averaged. The steps were repeated for the contralateral limb.

### 2.4. Data Analyses

For statistical analysis, SPSS version 25.0 was used. Initially, the sample was characterized through graphics and tables. Then, the arithmetic means of each dependent variable were calculated, namely the length, width, and thickness for each group, gender, age, height, dominant limb, and LPA, and were subsequently compared with each other.

Descriptive statistics were analyzed to evaluate the correlation between the variables under study. The last stage of this procedure was carried out using inferential statistics. Initially, we carried out the Shapiro–Wilk test and the Levene test.

Considering the results of the previous tests, the means were compared between groups using the ANOVA, Bonferroni post-hoc, and Student’s *t*-tests and, finally, the Pearson correlation coefficients between the variables under study. For all of these tests, the significance level was maintained at 5%, a value established for studies in the social and behavioral sciences.

## 3. Results

The sample consisted of 55.7% male individuals (*n* = 64) and 44.3% females (*n* = 51). Of the 115 participants, 34.78% were NSPs, 32.17% were cyclists, and 33.04% were volleyball players. Of the NSPs, 50% were female and 50% were male.

Table 1 displays the average values of the PT measurements obtained.

The results demonstrate that PT measurements of length, width, and thickness were higher in males. A Student’s *t*-test was performed, and it showed that TPT measurements of both lower limbs (0.003 < *p* < 0.044) and WPTL (*p* < 0.003) differ statistically significantly between genders. Based on Pearson’s correlation coefficient (Table 2), it was found that the PT dimensions of both limbs are correlated with the male gender (0.336 < r < 0.601).

According to the results, there is a direct correlation between an increase in the dimensions of the PT and participation in sports. A correlation was observed between decreased width and increased sports practice, with a directly proportional increase in TPT.

The ANOVA test (Table 3) demonstrated that the LPT and TPT of both limbs differed with the group under study.

Furthermore, the post-hoc Bonferroni test (Table 3) showed differences in the WPT and TPTR measurements between the NSP group and the cyclist and volleyball player groups (*p* < 0.05). In contrast, in the TPTL measurements, significant changes were noticed between the NSP and volleyball player groups (*p* < 0.05). When analyzing the average difference, the LPT is lower in the NSP group compared to the SPs.

Pearson’s correlation coefficient (Table 2) demonstrates that cyclists and volleyball players have increased values of LPT and TPT since there are statistically significant differences in the mean of these dimensions bilaterally between SPs and NSPs (*p* < 0.05).

Concerning the variable age, the ANOVA test (Table 3) established the difference between the measurement of WPTL and the age of the participants (*p* < 0.025). In addition, the post-hoc Bonferroni test (Table 3) detected differences in the measurement of WPTL when compared between the age groups 17–19 years and 23–25 years (*p* < 0.05). 

Regarding the average value, in the group 15–17 years, the WPTL values were higher compared to the age group 17–19 years. A statistically significant difference was observed between the LPT and the age of the participants (*p* < 0.05) (Table 2).

As for the height, the mean was 172 ± 8.54 cm. The results show an increase in the LPT directly proportional to height. Individuals with heights between 160 and 170 cm had a lower WPTR and WPTL. TPT increased exponentially with height. The Pearson correlation coefficient (Table 2) revealed that values of moderate-to-strong intensity in the LPT are directly proportional to height (0.520 < r < 0.601).

From the study sample, 34.78% of individuals had an LPA “Low” (50% for each gender), 5.22% were “Moderate” (83.3% of the female gender), and 60% were “High” (62.3% for the male gender). It was visualized as an increase in length directly proportional to the LPA. Despite this, there was no direct proportionality relationship between TPT and LPA.

As a result of the ANOVA test (Table 3), it is possible to state the existence of a proportionality correlation between all PT measurements and LPA. The post-hoc Bonferroni test performed (Table 3) indicates there were differences in the length, width, and TPT of both lower limbs between the “Low” and “High” LPA groups (*p* < 0.05).

Table 2 shows a weak-to-moderate correlation between the length and thickness dimensions with LPA in both limbs (0.281 < r < 0.422).

In this sample, there was a predominance of the right limb (91.30%; *n* = 105), and 18.4% of the volleyball players indicated the left limb. Left-handed subjects had a higher LPT than right-handed subjects, but regardless of the dominant limb, the LPTR was lower. Upon analysis of the data, the dominant limb had a higher LPT. Since TPTR values were similar, the only noticeable difference was a slight increase in TPTL.

Table 4 shows the descriptive statistics and the differences between cyclists and volleyball players. The results show significant differences between these groups. Volleyball players had higher LPT and TPTL values, while cyclists had increased WPT and TPTR.

There were also significant differences between genders. Values indicate that female cyclists have increased LPT, WPTL, and TPTL. Regarding the male gender, measurements were higher for volleyball players.

Towards age, a statistically significant difference stands out for cyclists from 15–17 years old in WPT and TPTR. For ages between 17 and 19 years old, there was an increase in LPT, WPTL, and TPT. For participants aged 19–21 years, the LTP and TPT values were higher in the group of volleyball players. All measurements showed a significant increase for volleyball players aged between 21–23 and 23–25 years old.

For height, we found significant differences in volleyball players except for WPTL, which was higher for cyclists.

In the dominant limb, an increase in LPT and TPT stands out for volleyball players on the right limb, while for the left limb, these differences are accentuated by increased WPTL and TPTR. On the other hand, in the “high” level LPA, cyclists had higher values for WPT and TPT.

## 4. Discussion

The results indicate that volleyball players have the highest LPT, and the NSP group has the lowest values. The level of correlation between the group and the LPT is weak. Therefore, there is an increase in LPT directly proportional to the study group. This result coincides with the Megías study [1], which showed a difference between NSPs and SP for the longitudinal axis (*p* = 0.015). The NSP group had the highest mean values of WPT, while the smallest WPTR measurements were observed in the volleyball player group and WPTL in the cyclist group. It should be noted that there is only a weak correlation (r = 0.248) between the WPTL and the study group. These results are contrary to those obtained by Giacchino and collaborators [12] because they obtained differences in the TPT (*p* = 0.165). In agreement with another study [1], it was concluded that there was a relationship between the groups of athletes and NSPs on the transversal axis (*p* = 0.005). The results indicate that individuals belonging to the cyclist group have the highest TPTR, while volleyball players have a higher TPTL. The lowest mean TPTR values were analyzed in the NSP group. There are weak correlations between the study group and the TPT, which translates into an exponential increase in this variable as a function of the PAL in each group. These results corroborate others [1] since a strong correlation of 98% was found between the TPT increase in both axes and the PAL, so it was concluded that regular physical activity leads to an increase in the PT size. Therefore, there is an increase in PT length and thickness bilaterally in SPs.

Regarding the relationship between LPA and PT measurements, it was found that individuals with “High” LPA have a higher mean value of LTP, with a moderate association. This result clearly shows that there is a direct proportionality between LTP and LPA, which means that an increase in LPA leads to an increase in LTP. The literature [1] concluded that the practice of regular physical activity leads to an increase in the size of the PT in healthy individuals. The highest mean WPT value was measured in individuals with a “Low” LPA. Contrary to what happens in the relationships between the other variables, between WPTL and NAF, the increase in WPT is inversely proportional to the NAF. The highest TPTL was evaluated in subjects with a “Moderate” LPA and the TPTR with a “High” LPA. After the comparative analysis of the TPT between the various LPA groups, it was concluded that this is a weak correlation for the right limb with r = 0.334 and the left with r = 0.281. This translates into an exponential increase in TPT as the LPA increases. These results support another study [14], in which it was noticed that TPT is higher in professional players (*p* < 0.05), so it is concluded that the practice of regular physical activity leads to an increase in the size of the PT in healthy individuals [1]. Therefore, there is a correlation among LPTR, LPTL, WPTL, TPTR, TPTL, and the performance of the physical activity.

Based on the analysis of the relationship between gender and PT measurements, it can be concluded that males have a higher incidence of PT. The LPTR has a moderate correlation (r = 0.587), while the LPTL has a strong correlation (r = 0.601). This result supports the study carried out by Carrol and collaborators [6], in which gender influenced LPT, being higher in men (*p* < 0.05). There is evidence of a weak correlation between gender and WPT. Regarding thickness, there is a moderate association between gender and TPT. The present research study concluded that PT measurements have a higher mean value in males. This result matches other studies [15], which support PT being thicker in males (*p* = 0.001).

It is essential to note that the highest LPT was observed in individuals with a height of 180 cm or more, concerning the dependent variables. Individuals with heights between 150 and 160 cm have the highest average width value, while those with heights equal to or greater than 1.80 m have the highest values of TPT. When comparatively analyzing the LPT means as a height function, moderate correlations are observed (r = 0.534 and r = 0.520). These results indicate an exponential increase between the LPT and the height of the individuals. As for the correlations between height and WPT, there is a weak relationship (r = 0.349 and r = 0.337). As analyzed in terms of length, the WPT also shows an exponential increase between width and height. The correlation between height and TPT was weak (r = 0.378 and r = 0.359) and showed an exponential increase between TPT and height. The authors of the literature [16] concluded that the LTP and TPT increase with height, which is consistent with our results.

The last assessment was related to the age group. The highest length values were obtained in individuals aged between 19 and 21 years. There were no differences in LPT measurements. Regarding this relationship, a study [6] argues that aging does not affect LPT. The highest values of width were evaluated in individuals included in the age group 23–25 years. There is evidence of a weak correlation between age and WPT (r = 0.254 and r = 0.284). This result demonstrates an increase in WPT directly proportional to the age group. Contrary to the results obtained in this study, Ferrando [17] found no correlation between WPT and age. The study found that the highest TPTR values were observed in individuals aged between 19 and 21 years old. On the other hand, the highest mean TPTL value was found in individuals aged between 21 and 23 years old. Similar to length, there were no differences in the correlation between this measurement and height or thickness. This result contradicts the information of Todd and collaborators [16], in which there is a correlation between TPT and age (*p* = 0.0007).

In this study, it was found that there is a correlation among gender, height, and PT measurements. However, the correlation between age group and PT dimensions only influenced WPT.

Regarding the dominant limb, the highest mean LPT is associated with left-handed individuals. The WPT was higher for the right limb belonging to right-handed individuals, while the left limb was in left-handed individuals. These results are consistent with two other recent studies [16,18] since the variation in length and width between the right and left leg was not statistically significant (*p* = 0.6) despite the trend of increasing TPT in the dominant (*p* = 0.095). No correlation was observed between the PT dimensions and the dominant limb.

Regarding the comparison between sports, the study results show significant differences. Volleyball players have higher LPT and TPTL values, while cyclists have increased WPT and TPTR. We also detected substantial differences between genders, with female cyclists increasing in LPT, WPTL, and TPTL and male measurements being higher for volleyball players. Towards age, a statistically significant difference stands out for cyclists aged 15–17 and 17–19 years and for volleyball players aged 19–21, 21–23, and 23–25 years. For height, we found significant differences in volleyball players, except for WPTL, which was higher for cyclists. In the dominant limb, an increase in LPT and TPT stands out for volleyball players on the right limb, while for the left limb, these differences are accentuated by increased WPTL and TPTR. On the other hand, in the “high” level LPA, cyclists had higher values for WPT and TPT.

The findings of the present exploratory study point out that volleyball players have a PT that is more developed compared with cyclists; however, it is important to promote passive stabilization of the PT because of knee movement during the practice of this sport. For example, volleyball players have the highest LPT, so the knee extensor mechanism, mainly composed of the quadriceps muscle and tendon, the patella, and the PT, must be pivotal to dynamically stabilize the knee during multiple valgus and rotatory stresses. Moreover, the landing phase of jumps involves an eccentric load on the PT to accurately control the braking of the lower limb on the ground.

Despite our study not evaluating PT pathologies, it is important to understand that musculoskeletal injuries are very common among sports practitioners, and detailed knowledge of biomechanical features of cycling and volleyball-specific gestures is pivotal to performing a detailed sonographic assessment of the affected anatomical site and planning a suitable rehabilitation approach.

Some limitations were the scarcity of ultrasound evaluation studies in the chosen sports and the inter and intra-observer variability of the ultrasound. To try to minimize this factor, measurements of the PT of each limb were performed three times by the researcher, using the average for the statistical analysis. Furthermore, measuring the length of the patellar tendon was a challenge because the linear probe only covered a 5 cm area, while most individuals have a longer PT, so it required sectional measurements. In the future, a standardized process should be established for measuring PT. If it is available, authors can use the panoramic mode of the ultrasound machine to avoid the use of a wire.

It is important to note that the non-probabilistic sampling method used in the study may not be fully representative of the population. Therefore, it is recommended to use a more representative sampling method, such as the probabilistic one, in future studies. Additionally, it is suggested to conduct further investigations with greater homogeneity between dominant limbs and sports practitioners among each group under study.

## 5. Conclusions

The PT measurements were analyzed concerning several factors, such as the group, gender, age, height, dominant limb, and LPA.

The study showed that the increase in LPT and TPT is directly proportional to sports practice. The highest mean values of LPT were evaluated for volleyball players, and the highest mean TPT values were equivalent for cycling and volleyball players. As for the width value, the highest value was analyzed in the NSP group.

Additionally, the study showed that gender influences all PT measurements, and the values were higher for the male gender, while age only interferes with WPT.

Furthermore, there was an increase in LPT and TPT that was directly proportional to the increase in height. However, there are no clear associations between dominant limb and PT measurements.

It was possible to conclude that sports stimulated PT, increasing LPT and TPT. The influence of LPA translates into an increase in LPT, TPT, and WPTL.

The results presented are relevant as far as they demonstrate and prove that numerous variables influence PT measurements. Besides that, they highlight the advantages of physical activity in promoting passive stabilization of the patellar tendon, which is more effective in preventing pathologies associated with the PT and patella.

## Figures and Tables

**Table 1:** Group, gender, age, height, limb-dominance, and physical-activity-level-related descriptive statistics of patellar tendon measurements in millimeters.

Variables		LPTR		LPTL		WPTR		WPTL		TPTR		TPTL	
		$\bar{X}$	*σ*	$\bar{X}$	*σ*	$\bar{X}$	*σ*	$\bar{X}$	*σ*	$\bar{X}$	*σ*	$\bar{X}$	*σ*
Group	Non-sports practitioners (*n* = 40)	48.07	1.16	47.71	1.17	25.73	0.45	25.46	0.40	3.20	0.09	3.09	0.09
	Cycling (*n* = 37)	55.71	1.57	55.12	1.57	24.84	0.57	24.29	0.43	3.70	0.12	3.51	0.10
	Volleyball players (*n* = 38)	55.97	1.54	56.19	1.62	24.45	0.44	23.77	0.50	3.69	0.12	3.52	0.12
Gender	Female (*n* = 51)	46.92	0.98	46.38	0.91	23.88	0.45	23.34	0.38	3.16	0.08	3.03	0.08
	Male (*n* = 64)	58.09	1.03	58.09	1.09	25.93	0.33	25.47	0.32	3.81	0.09	3.63	0.08
Age	15–17 years (*n* = 15)	51.37	2.21	51.25	2.21	24.26	0.89	24.02	0.66	3.46	0.19	3.36	0.21
	17–19 years (*n* = 24)	52.87	1.94	52.82	1.97	23.94	0.67	22.96	0.64	3.64	0.14	3.36	0.09
	19–21 years (*n* = 18)	55.64	2.67	56.40	2.38	24.82	0.52	24.83	0.65	3.70	0.20	3.29	0.13
	21–23 years (*n* = 30)	52.35	1.60	51.57	1.76	25.33	0.42	24.70	0.47	3.36	0.11	3.40	0.15
	23–25 years (*n* = 28)	53.55	1.84	53.01	1.95	26.16	0.66	25.75	0.47	3.51	0.14	3.39	0.13
Height	1500–1600 mm (*n* = 1)	43.70	----	46.10	----	26.20	----	26.77	----	2.60	----	3.13	----
	1600–1700 mm (*n* = 12)	44.81	0.92	44.71	1.13	22.64	0.85	22.98	0.46	3.19	0.10	2.98	0.15
	1700–1800 mm (*n* = 35)	47.82	1.38	47.29	1.15	24.07	0.49	23.16	0.48	3.20	0.11	3.10	0.11
	>1800 mm (*n* = 67)	57.55	1.03	57.39	1.14	25.92	0.34	25.48	0.32	3.76	0.09	3.58	0.08
Limb dominance	Right (*n* = 105)	52.68	0.94	52.45	0.97	25.07	0.30	24.45	0.27	3.52	0.07	3.34	0.07
	Left (*n* = 10)	57.95	1.71	57.62	1.83	24.50	0.91	25.30	1.09	3.53	0.19	3.66	0.16
LPA	Low (*n* = 40)	48.07	1.16	47.71	1.17	25.73	2.45	25.46	2.14	3.20	1.14	3.09	1.18
	Moderate (*n* = 6)	49.19	0.45	48.50	0.40	22.03	1.48	22.15	1.47	3.55	0.36	3.73	0.33
	High (*n* = 69)	56.42	0.09	56.29	0.09	24.87	0.27	24.19	0.53	3.71	0.09	3.49	0.07

Notes: length (LPT), right (R) and left (L), width (WPT), thickness (TPT), level of physical activity (LPA).

**Table 2:** Pearson’s correlation coefficient of both limbs correlated with gender, group, age, height, LPA, and limb dominance.

Variable		LPTR	LPTL	WPTR	WPTL	TPTR	TPTL
Gender	R	**0.587**	**0.601**	**0.336**	**0.374**	**0.449**	**0.450**
	Sig level	**0.000**	**0.000**	**0.000**	**0.000**	**0.000**	**0.000**
Group	R	**0.347**	**0.363**	−0.174	**−0.248**	**0.288**	**0.270**
	Sig level	**0.000**	**0.000**	0.063	**0.008**	**0.002**	**0.003**
Age	R	0.039	0.012	**0.254**	**0.284**	−0.055	0.025
	Sig level	0.676	0.901	**0.006**	**0.002**	0.556	0.794
Height	R	**0.534**	**0.520**	**0.349**	**0.337**	**0.378**	**0.359**
	Sig level	**0.000**	**0.000**	**0.000**	**0.000**	**0.000**	**0.000**
LPA	R	**0.420**	**0.422**	−0.118	**−0.199**	**0.334**	**0.281**
	Sig level	**0.000**	**0.000**	0.210	**0.033**	**0.000**	**0.002**
Limb dominance	R	0.157	0.151	−0.053	0.084	0.002	0.137
	Sig level	0.093	0.108	0.577	0.370	0.979	0.143

Notes: length (LPT), right (R) and left (L), width (WPT), thickness (TPT). Significant differences values are in bold.

**Table 3:** ANOVA and post-hoc Bonferroni test used to verify statistically significant differences among groups, age, and LPA in patellar tendon measurements.

ANOVA Test				Post-Hoc Bonferroni Test			
Variables		Z	Sig Level	Variables		Mean Difference	Sig. Level
**Group**	LPTR	9.118	0.000	NSP	Cyclists	−1.648	0.003
					Volleyball players	−1.912	0.000
				Cyclists	Volleyball players	−0.264	**1.000**
	LPTL	9.545	0.000	NSP	Cyclists	−1.804	0.002
					Volleyball players	−2.066	0.000
				Cyclists	Volleyball players	−0.262	**1.000**
	WPTR	1.608	**0.205**				
	WPTL	2.723	**0.070**				
	TPTR	8.387	0.000	NSP	Cyclists	−0.539	0.001
					Volleyball players	−0.508	0.003
				Cyclists	Volleyball players	0.031	**1.000**
	TPTL	4.058	0.020	NSP	Cyclists	−0.319	**0.107**
					Volleyball players	−0.400	0.025
				Cyclists	Volleyball players	−0.081	**1.000**
**Age**	LPTR	0.533	**0.712**				
	LPTL	0.751	**0.559**				
	WPTR	1.687	**0.158**				
	WPTL	2.894	0.025	15–17 years	17–19 years	0.258	**1.000**
					19–21 years	−0.089	**1.000**
					21–23 years	−0.067	**1.000**
					23–25 years	−0.283	**1.000**
				17–19 years	19–21 years	−0.347	**0.569**
					21–23 years	−0.325	**0.427**
					23–25 years	−0.542	0.011
				19–21 years	21–23 years	0.022	**1.000**
					23–25 years	−0.194	**1.000**
				21–23 years	23–25 years	−0.217	**1.000**
	TPTR	0.389	**0.816**				
	TPTL	0.192	**0.942**				
**LPA**	LPTR	10.695	0.000	Low	Moderate	−0.342	**1.000**
					High	−1.907	0.000
				Moderate	High	−1.565	**0.258**
	LPTL	11.550	0.000	Low	Moderate	−0.250	**1.000**
					High	−2.083	0.000
				Moderate	High	−1.833	**0.173**
	WPTR	4.187	0.018	Low	Moderate	0.825	0.015
					High	0.151	**0.747**
				Moderate	High	−0.674	**0.052**
	WPTL	3.527	0.033	Low	Moderate	0.650	0.038
					High	0.172	**0.427**
				Moderate	High	−0.478	**0.172**
	TPTR	8.627	0.000	Low	Moderate	−0.350	**0.669**
					High	−0.538	0.000
				Moderate	High	−0.188	**1.000**
	TPTL	4.035	0.020	Low	Moderate	−0.233	**1.000**
					High	−0.371	0.016
				Moderate	High	−0.138	**1.000**

Notes: length (LPT), right (R) and left (L), width (WPT), thickness (TPT). Significant differences values are in bold.

**Table 4:** Descriptive statistics of patellar tendon measurements of both limbs correlated with gender, group, age, height, LPA, and limb dominance between cyclists and volleyball players.

Variables		Group	Gender		Age (years)					Height (mm)			Limb Dominance		LPA	
			Female	Male	15–17	17–19	19–21	21–23	23–25	1600–1700	1700–1800	>1800	Right	Left	Moderate	High
**LPTR**	Z	0.002	1.877	1.734	10.762	0.887	0.055	0.313	0.001	1.626	0.452	0.158	0.304	3.325		0.010
	***Sig***	**0.966**	**0.181**	**0.195**	0.010	**0.357**	**0.818**	**0.588**	**0.980**	**0.249**	**0.510**	**0.693**	**0.584**	**0.111**		**0.921**
	***t***	−0.118	0.556	−1.480	1.414	0.331	−0.467	−0.371	−0.333	−0.705	0.062	−0.047	−0.055	0.541	−0.727	−0.374
**LPTL**	Z	0.207	0.143	0.056	12.649	1.118	0.062	0.222	1.564	3.620	0.350	0.407	0.766	2.247		0.231
	***Sig***	**0.651**	**0.708**	**0.814**	0.006	**0.302**	**0.809**	**0.647**	**0.232**	**0.106**	**0.532**	**0.527**	**0.385**	**0.178**		**0.632**
	***t***	−0.478	0.104	−1.486	1.454	0.193	−0.848	−0.298	−0.544	−0.409	−0.554	−0.228	−0.416	0.253	−0.621	−0.793
**WPTR**	Z	2.940	1.794	0.253	3.068	7.949	1.584	2.571	0.376	3.876	1.632	0.144	1.212	18.159		3.415
	***Sig***	**0.091**	**0.191**	**0.618**	**0.114**	0.010	**0.234**	**0.140**	**0.550**	**0.097**	**0.218**	**0.706**	**0.275**	0.004		**0.069**
	***t***	0.550	−0.134	0.671	0.772	0.719	1.882	−1.090	−0.376	−0.570	0.004	1.024	0.329	0.351	−1.282	0.472
**WPTL**	Z	0.591	0.457	2.744	0.366	1.491	0.480	0.069	0.237	4.735	0.961	2.095	0.107	0.690		0.351
	***Sig***	**0.445**	**0.505**	**0.105**	**0.560**	**0.236**	**0.503**	**0.798**	**0.634**	**0.072**	**0.340**	**0.155**	**0.745**	**0.434**		**0.556**
	***t***	0.785	0.908	−0.061	1.722	1.335	0.791	−0.345	−1.401	0.823	0.819	0.424	1.240	−0.359	0.123	0.461
**TPTR**	Z	0.150	0.050	0.092	0.651	0.497	0.000	6.743	1.167	2.253	0.292	0.058	0.267	4.567		0.135
	***Sig***	**0.700**	**0.824**	**0.763**	**0.441**	**0.489**	**0.997**	0.027	**0.298**	**0.184**	**0.596**	**0.811**	**0.607**	**0.070**		**0.714**
	***t***	0.012	−0.072	−0.256	0.349	1.721	−1.087	−0.800	−0.450	−0.856	0.507	−0.174	−0.019	−0.397	−0.531	0.028
**TPTL**	Z	1.104	0.258	0.849	1.204	1.007	0.191	8.751	0.237	3.282	0.025	1.592	1.031	1.682		0.002
	***Sig***	**0.297**	**0.615**	**0.362**	**0.301**	**0.327**	**0.670**	0.014	**0.634**	**0.120**	**0.877**	**0.214**	**0.314**	**0.236**		**0.961**
	***t***	−0.045	0.484	−0.830	−0.339	1.399	−0.797	−0.935	0.473	0.976	0.333	−0.641	−0.042	0.346	−0.572	0.430

Notes: length (LPT), right (R) and left (L), width (WPT), thickness (TPT), level of physical activity (LPA). Significant differences values are in bold.

## Data Availability

The datasets used and/or analyzed during the current study are available from the corresponding author upon reasonable request.
